# The Role of Family Members in Psychiatric Deep Brain Stimulation Trials: More Than Psychosocial Support

**DOI:** 10.1007/s12152-023-09520-7

**Published:** 2023-05-26

**Authors:** Marion Boulicault, Sara Goering, Eran Klein, Darin Dougherty, Alik S. Widge

**Affiliations:** 1 Department of Philosophy, University of Edinburgh, Edinburgh, UK; 2 Center for Neurotechnology, University of Washington, Seattle, WA USA; 3 Department of Philosophy, University of Washington, Seattle, WA USA; 4 Department of Neurology, Oregon Health & Science University School of Medicine, Portland, OR USA; 5 Neurotherapeutics Division, Department of Psychiatry, Massachusetts General Hospital, Boston, MA USA; 6 Harvard Medical School, Boston, MA USA; 7 Medical Discovery Team on Addiction, University of Minnesota, Minneapolis, MN USA; 8 Department of Psychiatry & Behavioral Sciences, University of Minnesota, Minneapolis, MN USA

**Keywords:** Deep Brain Stimulation, Family members, Caregivers, Relationships, Dyadic analysis, Deep Brain Stimulation for psychiatric conditions, Identity, Medical decision-making

## Abstract

Family members can provide crucial support to individuals participating in clinical trials. In research on the “newest frontier” of Deep Brain Stimulation (DBS)—the use of DBS for psychiatric conditions—family member support is frequently listed as a criterion for trial enrollment. Despite the significance of family members, qualitative ethics research on DBS for psychiatric conditions has focused almost exclusively on the perspectives and experiences of DBS recipients. This qualitative study is one of the first to include both DBS recipients and their family members as interview participants. Using dyadic thematic analysis—an approach that takes both the individuals and the relationship as units of analyses—this study analyzes the complex ways in which family relationships can affect DBS trial participation, and how DBS trial participation in turn influences family relationships. Based on these findings, we propose ways to improve study designs to better take family relationships into account, and better support family members in taking on the complex, essential roles that they play in DBS trials for psychiatric conditions.

**Supplementary Information:**

The online version contains supplementary material available at 10.1007/s12152-023-09520-7.

## Introduction

Psychiatric conditions are the leading cause of disability worldwide [1]. Estimates suggest that for over 20% of those living with major depressive disorder (MDD) and obsessive-compulsive disorder (OCD), conventional treatments, such as medication and talk therapies, have limited efficacy, and relapse rates are high [2–4].

In search of more effective treatments, some researchers have turned to Deep Brain Stimulation (DBS), an intervention widely used to treat motor disorders such as Parkinson's disease [5, 6]. Using electrodes implanted deep in the brain and a pulse-generator device (similar to a heart pacemaker) implanted under the collarbone, DBS delivers electrical stimulation to brain targets. The specific target depends on the condition being treated; for example, DBS for essential tremor typically targets the ventral intermediate thalamus [7]. First employed in the 1950s as a tool for the localization of brain structures for ablation, DBS has now been used to treat movement disorders in more than 100,000 patients worldwide [7–9]. 

Since the first study of DBS for OCD in 1999 [10], trials of DBS for psychiatric disorders—dubbed “the newest frontier for DBS” [5, 11] —have yielded mixed though promising results [12–18]. Yet DBS also raises ethical issues related to, for example, privacy and access to neural data, and to the potential effects of DBS on an individual’s sense of personal identity, autonomy, and agency [19, 20].

Findings from early qualitative ethical studies point to the significance of *social relationships* for understanding and assessing the ethical implications of psychiatric DBS. In a 2015 study, psychiatric DBS recipients reported that family members frequently noticed changes in their symptoms before they themselves did [21]. A 2016 focus group study of 15 DBS recipients identified “relationship effects” as one of four central themes [22]. These findings are particularly significant given (a) the fact that family member support is frequently an inclusion criterion for DBS trial participation [11], and (b) the existence of ethical debates about the appropriate role of family members in clinical trials (e.g. debates about how to involve family members while preventing coercion or manipulation of patients), especially when trials involve “vulnerable populations,” such as those with psychiatric conditions [23, 24]. Research exploring the relational dimensions of psychiatric DBS thus has practical and normative implications for trial design, especially concerning questions of justice and access to trial participation, and may also contribute to longstanding bioethical questions about autonomy and family involvement in medical decision-making.

Qualitative research on the lived experiences and perspectives of DBS recipients is crucial for understanding the relational dimensions of DBS and its ethical implications. But, as some bioethicists have demonstrated, engaging family members in this research allows for deeper and more impactful analysis. For example, Thoresen and Lillemoen's [25]’s inclusion of both patients and their relatives as participants in their study of advance care planning in Norwegian nursing homes enabled them to understand how patients and relatives often functioned as what they called an “intertwined unit” in medical decision-making, which, they argued, has implications for end of life care. Yet, despite the significance of family member perspectives, qualitative research on DBS for psychiatric conditions has focused almost exclusively on DBS recipients [21, 26, 27].

This study is one of the first to interview both psychiatric DBS recipients and their family members (see Thomson et al. [28] for another such study). It is also the first that we are aware of to analyze the interview data using dyadic methods—i.e. methods aimed at analyzing the perspectives and experiences of participants both as individuals and as members of dyadic relationships—which, as discussed further in the Methods section below, allows for more robust relational analyses [29]. Using these methods, we identify six themes concerning how relationships mediate experiences of DBS for psychiatric disorders and, conversely, how DBS affects relationships.

## Methods

### Recruitment and Participant Information

Participants were recruited from a registry of individuals (*n* = 23 at the time of recruitment) receiving DBS for psychiatric disorders at a major US research hospital. DBS recipients were eligible if they (a) had a diagnosis of either MDD or OCD; (b) had a DBS device implanted; (c) were between the ages of 18 and 75; (d) were able to provide informed consent; (e) were an English speaker; and (f) had a close family member, loved one or care partner involved in their daily life also willing to participate in the study. Family members were eligible if they (a) were identified by the DBS recipient as a close family member, loved one or care partner involved in their daily life; (b) were between the ages of 18 and 75; (c) were able to provide informed consent; and (d) were an English speaker. Twelve DBS recipients met the eligibility criteria and were contacted by email or telephone and 7 chose to participate, along with 7 family members. Family members were selected and recruited using information provided by the DBS recipients.

Institutional Review Board (IRB) approval was granted on May 28, 2019 (the reviewing IRB was at the major US research hospital where participants were recruited). A consent form was sent to participants at least a week in advance of the interview, and a phone call was made to discuss the consent form and answer any questions. For phone or online video interviews, consent forms were signed and returned in a stamped self-addressed envelope provided by the study team. For in-person interviews, the consent form was signed in-person before the start of the interview.

In what follows, DBS recipients are identified using a number, e.g., ‘1,’ and their family members are identified using that same number followed by the letter ‘F,’ e.g., ‘1F.’ At the time of interview, all DBS recipients were undergoing DBS targeted to the ventral internal capsule/ventral striatum (VCVS) region of the brain and all had a primary diagnosis of either treatment-resistant OCD (*n* = 3) or MDD (*n* = 4). All DBS recipients with a primary diagnosis of OCD were diagnosed with MDD as a co-occurring disorder, and one was also diagnosed with Bipolar 1 and ADHD. One patient with MDD was diagnosed with PTSD as a co-occurring disorder. Individuals at these stages of illness typically have tried standard evidence-based therapies, including medication, neuromodulation therapies (such as electroconvulsive therapy and transcranial magnetic stimulation) and psychotherapy (such as cognitive-behavioral therapy and exposure–response therapy). Socioeconomic background varied; for example, 1 and 1F described their financial dependence on disability payments, whereas 6 and 6F were a high-income couple. Demographic profiles of the DBS recipients interviewed are shown below in Table 1.

**Table 1:** DBS recipient demographic information

Sex	# (%)
Male	4 (57%)
Female	3 (43%)
Nonbinary	0 (0%)
Race/ethnicity	
White or Caucasian	6 (86%)
Hispanic or Latino	1 (14%)
Black or African American	0 (0%)
Asian	0 (0%)
American Indian/Alaska Native	0 (0%)
Age	
25–34	2 (29%)
35–44	0 (0%)
45–54	1 (14%)
55–64	2 (29%)
Over 65	2 (29%)
DBS recipient-family member relationship	
Spousal	4 (57%)
Long term partnership	1 (14%)
Parent–child	2 (29%)
Primary diagnosis	
Major Depressive Disorder	4 (57%)
Obsessive Compulsive Disorder	3 (43%)
Years since initial DBS implantation (at time of interview)	
14 years	1 (14%)
11 years	3 (43%)
9 years	2 (29%)
6 years	1 (14%)
Co-occurring disorders	
Post-Traumatic Stress Disorder	1 (14%)
Major Depressive Disorder	3 (43%)
Bipolar 1 Disorder	1 (14%)
Attention Deficit Hyperactivity Disorder	1 (14%)
None	3 (43%)
DBS outcome (as assessed by DBS recipient at time of interview)	
Highly successful	2 (29%)
Moderately successful	4 (57%)
Minimally successful	1 (14%)

### Data Collection

Seven pairs of interviews were conducted, one with each DBS recipient and a separate interview with a family member of each recipient, for a total of 14 interviews. An interview guide with open-ended questions was used to structure the interviews, which were audio-recorded. The interview guide included questions on the relational dimensions of the following topics: participants’ understanding of DBS, informed consent, decision-making, expectations, identity, and agency (see Online Resource 1 for interview guide). There are different approaches to data collection for dyadic analysis—separate interviews, joint interviews, or some combination of the two (see Table 1 of Eisikovits and Koren [29] for a full list of approaches)—each of which offers different benefits and drawbacks. Given the sensitive nature of our interview topics, we chose to conduct separate interviews since this allows each participant to describe their experiences without having to consider the reaction of their family member when voicing criticism or bringing up sensitive topics [30]. To facilitate dyadic analysis (dyadic analysis is discussed in more detail below) we included questions intended to probe participants’ understanding of their loved one’s perspectives: for example, when we asked DBS recipients about their pre-implantation expectations, we also asked them what they thought their family members expected (and vice versa). Eight interviews were conducted in person in a conference room at the university hospital, 4 were via online video calls, and 2 were conducted on the phone. Interviews lasted an average of 71 min with a range of 28 to 94 min. The interviews were conducted by MB and took place between August 2019 and March 2020.

### Analysis

Interview audio recordings were transcribed verbatim by a professional transcription service and edited for accuracy by MB. Transcripts were de-identified and entered into Atlas.ti (Version 8.4.5) and analyzed using dyadic thematic analysis. Thematic analysis is a method that uses data coding practices to identify, analyze and report patterns, known as themes, within qualitative data [31]. The process involves highlighting and labelling (i.e. coding) significant quotes that relate in some way to the study’s research focus. These codes are then cross-analyzed and grouped to form broader themes [31, 32]. Three authors (MB, SG, EK) along with a research assistant were involved in coding the interview transcripts. Each coder coded 4 of the transcripts and all met to discuss and compare codes, resulting in a codebook with 265 codes. MB then used the codebook to code the remaining 10 transcripts. Themes were identified by analyzing the coding results alongside field memos written throughout the data collection process. Given the relatively broad goals of the study (to investigate the relational dimensions of DBS), we adopted an approach to thematic analysis that lay between what’s known as an “inductive approach” (where the research questions and concepts of interest evolve to some extent during the analysis process) and a “theoretical approach” (where coding is more driven by specific research questions and concepts) [31].

In contrast to conventional thematic analysis, dyadic thematic analysis takes as the unit of analysis each individual participant as well as “the meaning, perceptions of reality, and sense-of-being experienced by a dyad” [33, 186]. Initially the process is similar to conventional thematic analysis as described above: coding and determination of themes. However, for dyadic thematic analysis, each interview transcript is coded in two different ways: first as an independent interview, and second, alongside the transcript of their family member, with the aim of identifying overlaps and differences between their perspectives and experiences. In many instances during our analysis, the use of a dyadic approach altered our understanding of the meaning and significance of quotes from individual transcripts, leading to code updates. This process continued, going back and forth between updating codes at the individual and dyadic levels of analysis, until a set of key themes were determined [29].

Our approach to thematic analysis was grounded in a constructionist research epistemology that takes data and interpretations of data to be co-constructed in the process of research (in contrast to a realist research epistemology, which understands the goal of research to be the reflection of a reality independent of the researcher and the research process) [31, 34]. According to this research epistemology, the backgrounds, assumptions, characteristics, skills and perspectives of the researchers influence and co-construct how the interviews are conducted and how the data is coded and analyzed. For example, at the time of the interviews the interviewer in this study (MB) was based at an elite, high-resourced university and trained in empirical bioethics, which likely had an impact on the language and tone they used in the interviews and therefore on the kinds of responses elicited from the participants. At least one researcher on the team had personal experience with psychiatric disorders in their own family, leading them to understand some of the participants’ experiences in ways they may not otherwise have been able to.

## Results: Six Themes

Dyadic thematic analysis of audio transcripts yielded 6 themes salient to the relational dimensions of DBS (Table 2). The first theme encompasses how DBS recipients and their family members conceptualize the purpose of DBS. Themes 2 and 3 relate to different stages of participation in a DBS trial: the decision to enroll in a trial, and the process of understanding and navigating personal identity following implantation, respectively. Theme 4 relates to how the experience of undergoing DBS influences relationships, and in turn, theme 5 relates to how relationships influence experiences. Theme 6 concerns the values and priorities of family members.

**Table 2:** Themes

1. Conceptualizing DBS: DBS as one tool in a toolbox	DBS recipients and family members did not conceptualize DBS as a cure for psychiatric disorders, even when the outcomes were perceived to be highly successful. Instead, DBS is conceptualized as one “tool” within a broader therapeutic toolbox
2. Relationships and decision-making	DBS recipients described the decision to enroll in a DBS trial as a “last resort,” while also suggesting that family members played key roles in decision-making
3. Relationships and identity	DBS recipients described a difficult period of adjustment to their post-DBS selves. Family members played key roles during this period of adjustment
4. Effects of DBS experiences on relationships	Enrollment in a DBS trial had largely positive effects on family relationships. However, even when DBS was perceived to be successful, DBS also introduced new relational tensions related to trust and autonomy
5. Effects of relationships on DBS experiences	During a DBS trial, family members provided three kinds of support: (1) material support, e.g., financial assistance; (2) psychosocial support, e.g., mitigating the effects of stigma and (3) epistemic support, e.g., insight into how the device is affecting their loved one and whether DBS settings should be adjusted
6. Family member values and priorities	Family members valued being included in a DBS trial as a “team member,” and expressed a wish for greater access to data, as well as more post-trial support and information

### Conceptualizing DBS: DBS as One Tool in a Toolbox

DBS recipients all reported varying degrees of successful outcomes, where a successful outcome is defined as a reduction in psychiatric symptoms.^1^ Two reported highly successful outcomes (“I'm sorry, I get choked up. It worked…. it was a true gift” (6), “This has been a tremendous success for me” (2)). Four reported moderately successful outcomes (e.g., “I wish that the ability to do things up to my current full capacity, whatever that is, would last longer” (3)). One reported minimal success (“I've had on and off periods of success” (5)). Despite these largely positive outcomes, there was broad consensus among DBS recipients and their family members that DBS is “not a cure” but rather one “tool” within a wider therapeutic toolbox for managing psychiatric disorders:

“The DBS, the cortical leads, and the deep leads, they just are infused together to help me focus away from my OCD… but you still have to use cognitive behavioral therapy, too... It's not like it changes, like you're just 100% better.” (7)

“It's made a huge difference. I mean, it's not a cure but it allows me to be in a good mood, which is very handy now that I have five grandchildren. It allows me to be with people for an hour and a half, two hours, which is also good, and to go out places. I still have pretty constant anxiety and trouble, but I take things for that. I mean, it's under control and it's stable.” (3)

“And that's the thing I've learned about DBS, is it doesn't solve everything… And that was a tough lesson for me to learn.” (6F)

Clinical therapies were not the only kinds of tools in the therapeutic toolbox. As we’ll see in theme 5 below, family support (of multiple kinds) served as a therapeutic tool. Additionally, one family member believed that her husband’s attitudes – namely his self-motivation, belief and trust in his DBS – worked as a kind of therapy alongside the DBS stimulation:

“Because he is motivated to get the best out of something… then he puts his trust in it. He believes DBS works, and then because he believes it works, he pushes himself to do certain things.” (3F)

### Relationships and Decision-making

DBS recipients and their family members saw the decision to enroll in a DBS trial as pivotal. Most used fatalistic language to describe the decision-making process, frequently referring to DBS as a “last resort.” Some described feelings of urgency and desperation, while others described apathy and hopelessness. For the majority, the decision did not feel like a decision at all: enrolling in a DBS trial felt like the only option available.

“It was the only decision. There really weren't other choices.” (1)

“I definitely think it was the right decision, but also I would say we didn't really have any option… We had done the ECTs, and the magnetic thing, and he was on a lot of medication. So, in my mind, we had really nothing to lose except that he might have a crisis in surgery.” (3F)

“But at that point, to be honest, I really didn't care…This was going to be my last step... I had no hope, I really didn't care about anything.” (6)

“It was in a sense, why not do it? The alternative of not doing it really wasn't a good alternative.” (4F)

“I'm ready to try anything. I don't care whatever it is.” (7F)

While interviewees believed that there was no “real” decision to be made, they nonetheless identified two kinds of roles that family members played in the decision-making process. First, relationships and family members served as a *motivation* for enrolling in a DBS trial:

“Oh, to be honest with you, on a conscious level, the relationships were the primary thing. The only reason I was still alive was because I didn't want my wife and my daughter to have to live with their father and their husband killing himself.” (2)

“Yes, I think it was the right decision because for me, I could have not gotten a DBS and been disabled my whole life, or... getting the DBS at least gives me sort of a chance to have a normal-ish life… and I think that's what my mom would want for me.” (5)

“[H]e was motivated to do something for himself. But I think that I push him to do the most with who he can be.” (3F)

In a second role, family members served as *partners* in decision-making. Partnership came in multiple forms and degrees, including assisting DBS recipients with decision-making tasks from reading consent forms or doing research on DBS, to participating in “processing” the decision, to collaborating in “problem solving,” to deliberating about and agreeing upon the final decision itself:

“I don't think I really processed [the decision] with anybody except my wife.” (2)

“We discussed it, and I think it was more the risk of the brain surgery… But, after everything that 2 has been through, and for the length of time that he's suffered with depression and mental illness, based on what the doctor told us, what research we did… based on all of those facts, we decided to go ahead with it. Both of us.” (2F)

“I was the decision maker. He was 20, so... And he wasn't capable of doing any kind of research or processing any of the information, so I made it clear to him that it was his choice, but I also let him know that this is what I recommended and Dr. X recommended, and his psychologist was in agreement.” (5F)

“It's a part of our relationship to do sort of joint problem solving about his life and his needs and his illness.” (5F)

This collaboration in decision-making extended to ongoing decisions about adjusting DBS settings. 6F gives an example of his role during study visits with the research team. During visits, a research team member tests different settings and asks for feedback from the DBS recipient. 6F would challenge his wife to try harder when he believed she was becoming fatigued and therefore no longer answering accurately:

“I've known her for 30 years so I could see her sort of giving up. Like she was like, "Sure. Better." And I was like, "No. This is the setting they're going to land on. So is it really better or is it not better?” (6F)

Although family members’ involvement in decision-making was largely described in positive terms, this involvement was not always tension-free. For example, 6F described how he felt like he sometimes inadvertently took control of study visits:

“I think she was also at this weird state where she was not thinking very clearly and had real trouble expressing her concerns or thoughts… And so I had a nasty habit of translating for her, which I'm sure pissed her off. But it also meant the doctors were talking more to me even though we were sitting next to each other and she was even asking a question.” (6F)

### Identity

DBS recipients reported changes in identity following DBS, some attributing them to the stimulation (“I’ve become more like my true self” (4)), and others to the kinds of changes that inevitably occur with time (“my own opinion is that we change all the time” (3)). Although these changes were described as either positive and/or expected, many DBS recipients nonetheless expressed difficulty in adjusting to them:

“Finding out who I am, whatever I am, at that stage but then I was going through that all along, kind of figuring myself out... I think that's the hardest thing.” (3)

“No one tells you how to restart your life all over again. I think I struggled with that for a long time, like, how do you go from negative 50 back to square one. I think that I don't know if they have rehabilitative programs or something, but I think it was really hard to navigate life after having been completely off the grid for two plus years.” (6)

Family members played a significant role during these periods of adjustment. For example, 2F describes how she works to “remind” her husband of what it’s like to live with a sense of self-worth, and to understand and endure “normal stresses,” and 6F describes directly engaging with his wife in conversations about identity change:

“And, because a lot has to do with self-worth, his idea of how he sees the world, how he sees himself, how he sees how people see him, and all of those weaknesses that present themselves to him. And I think with DBS, all that's gone away. So, he hadn't had to really learn what it's like to have normal stresses in your life. And I've had to remind them of that.” (2F)

“She and I have had some conversations where it gets to pretty deep stuff of which is the real her, the one on the batteries or before and this whole, ‘I'm a cyborg?’ Which is a very deep dark path and an interesting debate.” (6F)

### Effects of Relationships on DBS Experiences

Relationships were found to have significant effects on individuals’ experiences with DBS. These effects resulted from three kinds of relational support: (1) material support, (2) psychosocial support and (3) epistemic support. Material support includes financial, cognitive and logistical support needed for navigating life with a severe chronic illness while participating in a complex clinical trial. Interviewees described how their need for this kind of support decreased as psychiatric symptoms improved.

“I couldn’t take care of myself, so just right off the bat now, my dad helped me with the medication, with the DBS. I’d say, less than a year ago, my dad would drive me here, and he would park down the street and just say, ‘Call me when you’re ready.’… He used to give me my medication every night, my cocktail of medication… with DBS, now he doesn’t play a role in it... A few months ago, maybe less than that, I started driving around myself.” (7)

“The time when I had the hallucinations, he was great… He kind of took control and said, ‘Give me the phone number, I’m going to call, I’m going to set up an appointment, you’re going to be okay. We’re going to drive down today.’” (1)

“When I had the [DBS-related] infection, my mom took that time off... because I couldn’t hold my son.” (6)

In terms of psychosocial support, interviewees described how family members provided motivation, love, and consolation through difficult moments. One DBS recipient described how her son’s acceptance of DBS as normal helped her in “rejoining life” post-DBS, and one family member talked about how she would mitigate stigma by appealing to a sense of “normal for us:”

“I think my son thinks everyone has batteries in their chest. It’s funny, when he was little, he would drag the charger and I’m like, ‘We shouldn’t be playing with that right now.’ But I think it’s like rejoining life.” (6)

“I’m fond of saying, ‘what’s normal for us’ and it’s not normal for many families or marriages, but it's normal for us, and that's okay.” (3F)

Family members also play a role in identifying and understanding the effects of DBS; a type of support we term *epistemic support*. This finding arose when analyzing interviewees’ responses to a question about who is in the best position to judge whether DBS settings need to be adjusted. Some DBS recipients (1, 3 and 5) saw themselves as the best judge:

“Me, because I can tell how I feel and what I'm thinking… 1F doesn't actually have any idea.” (1)

“Well, I can do kind of a body and emotional inventory of what I'm feeling and if I'm just not getting the support ... I don't know how to describe it, but I can feel it.” (3)

“I'd have to kind of educate them [family members] on what's going on in my brain... in order for them to be able to notice if I'm having a thought that is a ‘[Participant 5] thought’ versus some other thought that's something else.” (5)

In contrast, 2 believed that his family members would be better judges because their perspectives are “more objective” than his own. His wife (2F) concurred, suggesting that she can see differences in 2 that he can’t see himself:

“Well, their [family members’] perspective is, I guess you could say, it could be more objective than my experience.” (2)

“I've seen a difference in Participant 2 in his confidence level, how he holds himself, how he's in the world, more confidence. But I don't think he sees that. And I think that's an underlying issue with him.” (2F)

1 expressed a similar sentiment regarding his father’s insights into his symptoms:

“My dad says, ‘Oh he's a whole other person.’ And that's… before I even start feeling real relief.”

6 believed that she and her husband as a couple are the best judges. However, she can also feel annoyed when her husband shares his perspective, despite the fact that she believed his opinion was usually “right” (more on this point in the discussion of Theme 5 below):

“I think it's probably a combination. I think it would be me initially with my husband a very close second.”(6)

“He might notice the cricking of the neck, or he'll say, ‘You seem agitated,’ which always annoys me. I'd say like 75% of the time, he's usually right if there's an issue. But, if it was really a problem, I think I would know… There were little nuances that I think I noticed that it would be hard for someone else to experience, plus he was working all the time… But I'd say it would be a combo. I'm going to give myself a little more credit on that front only because it's my body, but he'd be right there.” (6)

6F also reported engaging in more formal monitoring practices. He describes taking notes on 6’s symptoms and secretly checking her DBS controller at night to make sure her DBS is on:

“I probably have a ton of notes that were started. Like, ‘Here's my tracking, was it a good day or was it a bad day? Did we go in the car? Did we not go in the car? Where are we, at the airport?’ So that I could remember and then I would... I've actually secretly taken her controller and checked while she's asleep just to make sure it's on.” (6F)

Finally, we found that, in some cases, participants’ experiences of DBS changed when their family members shared their observations with them. Consider 2’s description of what happened when his family members told him that his symptoms had improved. Up until that point, he had not realized his symptoms had improved, and because of this, he hadn’t yet experienced the benefits of reduced symptoms. His family’s observations were themselves “healing”:

“And, you know, during the journey, each one of them has mentioned at times, ‘Boy, you really seem to be smiling a lot more. You really seem to be enjoying yourself more.’ My daughter has mentioned that to me a few times. And that in itself is kind of healing in a way. Because, she and my wife, when they say those things, they were right, but it's like it hadn't caught up to me yet. I hadn't realized that yet, because I was in such a... negative mindset to begin with.” (2)

### Effects of DBS Experiences on Relationships

The connection between relationships and DBS experiences goes both ways: just as relationships influence DBS experiences, so too do DBS experiences influence relationships. We identified a number of different mechanisms by which the latter occurs.

When DBS was successful the effects on relationships tended to be positive. In many cases, both DBS recipients and family members felt that the reduction in psychiatric symptoms allowed for reconnection and closer relationships:

“We're much closer…So the relationships have improved tremendously since the DBS occurred.” (2)

“I think the DBS has given my life a renewal of my life, the last 10 years. Because I've had the opportunity to really reconnect with 2 in day-to-day stuff, that we could never do before. Because he was in bed or he wasn't feeling well.” (2F)

“I can be closer to good friends than I could be before… I think my folks... I don't know who's more tolerant of who. I don't know if they're more tolerant of me, or if I'm more tolerant of them. There's been less conflict.” (1)

Relatedly, interviewees described improvements in mutual understanding and communication. 7F described how his son’s OCD symptoms made it difficult to communicate with him prior to DBS, and how this has changed:

“He would [prior to DBS] throw something if you say something really bad. Not really bad, if you say this is all whatever you are thinking, whatever is in his hand he will throw that from his hand… I can say a lot more to him now than I did before. Before, I wouldn't say anything to him.” (7F)

2F likewise described improvements in communication, while also noticing a shift in *relationship roles*. Prior to DBS, she took on the full “burden” of managing daily life. Since DBS, she can rely more on 2, and no longer feels she has to carry this burden alone:

“Well, he's been able to do more things. I've been able to communicate with him more, have more conversations with him, rely on him more for daily things, not have to carry the burden solely in our relationship.” (2F)

1 doesn’t believe her relationship with her partner would have been able to continue without DBS. This is in part because DBS enabled her (who was previously largely a receiver of care) to *take on an essential role as a caregiver* for her partner, who suffers from medical conditions himself:

“Could I have stayed [in the relationship] without the DBS? No. It's too hard. I wouldn't have been able to take care of both of us, and as it is I'm barely able to do that…” (1)

Not all of the effects of DBS on relationships were positive. 5, for whom DBS was only minimally successful, experienced frustration when his views on the success of DBS didn’t align with those of his family members and clinicians. Nonetheless, he understood that the expression of these contrary viewpoints was in his best interests:

“If I think I'm doing well but my therapist or my mom doesn't think I'm doing well... it's frustrating. But, so I probably don't really like it. But the thing is, it's actually good that they're being honest because mainly, they're actually helping me.” (5)

In other cases, the success of DBS improved existing relational dynamics, but also introduced new relational tensions, often resulting from the introduction of new relationship roles. 6 describes how during significant life events post-DBS implantation—such as the birth of her child—family members would question her ability to cope by herself. While 6 saw herself as capable of taking on a new caregiving role, her family members saw her as still occupying the “sick” role:

“I remember right after my son was born, my husband had to go away for a week and my mother stayed at the house because I think people didn't think I was capable of being home with a newborn, which was fine. But I was pretty hurt by that in a way because at that point it had been seven years... I think I had demonstrated that I was doing pretty well. So, I just felt like it was not a vote of confidence for me that someone had to stay with me...” (6)

Participant 3 describes a similar situation with his in-laws:

“My in-laws have had some suspicions at times and have been a little harder to settle down. I'm [3] not going to drive while the kids are in the car. They [the kids] are going to be put in the seat belts correctly, the car seat, whatever, et cetera.” (3)

These kinds of relational tensions were frequently expressed via questioning about whether the DBS is turned on or working. As discussed in Theme 4, family members’ observations provided unique insights into the effects of DBS (insights unavailable to DBS recipients by themselves), sometimes even influencing the effects themselves. However, these observations also had the potential to create tensions. Participant 1 describes her frustration in response to persistent questioning from her partner:

“He will say something to me about it, and I'll check, I'm good. I'm sorry you didn't like that I just chewed you out for something, but it's not the mood disorder. I'm on, I'm charged enough, and I just think you're an idiot.” (1)

In another example, 6 describes how her husband reacted to her expressions of agitation and stress by questioning whether the DBS was turned on. To her, this kind of questioning felt like “an attack.” She also highlights a relational asymmetry that DBS introduced: when her husband is agitated, she can’t question the authenticity of *his* feelings by asking whether *his* DBS is on:

“But we were moving, we had a horrible sale process, and I was just irate… So, I think I'm allowed to be agitated if there's a good reason... I can't shoot back at him. Well, is your device on? Because he didn't have one, but I know it just feels a little bit like an attack.” (6)

Family members also brought up the tensions that arose from this kind of questioning. 6F gave the following account of one such incident. In contrast to 6, though, he described this incident as “slightly funny”:

“There's a really interesting dynamic that's slightly funny, but she can also just be in a bad mood or have a bad day even though they're functioning. And I am very quick to say, ‘Are your devices off?’ And she's like, ‘No, I'm sure they're on.’ I'm like, ‘Check.’ And she's like, ‘No, they're on.’ And I'm like, ‘Check.’ And I'll make her get the thing and check and then they're on... I'm like, ‘Okay, so you're just being a bitch?’" (6F)

### Family Member Values and Priorities

As evidenced by the above findings, family members play multiple roles in DBS trials. This final theme encompasses family members’ values and priorities with respect to these roles. First, many family members described how much they value being included as members of what they see as a “team” charged with helping their loved one. For example, 5F describes how her inclusion within a research team (she uses the term “we” to describe decisions regarding DBS) was the “best part” of 5’s participation in a trial, providing a kind of treatment safety net, and significantly easing her caretaking burdens:

“The team has really been the best part for me is probably the main point… Having the team… I know if there's anything else that could help him, they will know. It's not up to me to be constantly doing the research anymore… I don't ever have to feel like, ‘What else can I do?... I'm not doing everything we could do.’ I know we are.” (5F)

This desire to be included as a team member relates to a desire expressed by 6F regarding access to data. He believes that “we could learn so much more if we had better access” and expresses frustration that the research team wasn’t sharing this data with him:

“I just felt like we could learn so much more if we had better access to all that stuff. And the stock answer from the doctors was, "You need to keep a blog or a log of every day how she's doing." And look, I would start that every time and then after a day and a half, you just don't do it or things are going well and you're like, ‘Oh great, I'm not going to do it anymore.’ And then lo and behold we'd have an issue.” (6F)

“So we'd go in the following week and they'd say, ‘Yes, it was off.’ And I'd say, ‘Okay, well when did it turn off?’… And they're like, ‘The device doesn't tell us, it doesn't keep all this data.’ And I'm like, ‘That's crap.’... Or ‘There was data, but it resets itself every day.’ Or they wouldn't give us the passwords.” (6F)

The family members we interviewed were all legally defined as family, with one exception. 1F is the long-term partner of 1, but they are not legally married. He believed that this led to him not being treated as a true “member of the team.” He suggested that research staff should draw on the idea of a “functional family” in deciding who to treat as a family member:

“I think there are times when we need to address the family, those who represent the family for the patient, not necessarily who are the family of the patient, but who are the functional family for the patient.” (1F)

Family members also frequently brought up confusion and anxieties about what would happen after the trial ends, particularly regarding post-trial responsibilities for continued care and medical support, questions concerning future financial costs of device maintenance and care, and what would happen if the trial didn’t result in FDA approval for DBS. One family member (3F) believed that post-trial financial issues were more of a concern for them than for their family member receiving DBS:

“It's also like what the hell happens 20 years from now, when something goes wrong... So that's something even though we were guaranteed by Medtronic, the company that makes this, that you [2 and 2F] will be helped.” (2F)

“And we were told that for whatever it was, a number of years things will be covered… [but] I always was a little hesitant… just wondering if we were going to be in this for a lot of money later, and it didn't affect our decision, but 3 was much less concerned.” (3F)

“I was a little concerned with where we were going to get appropriate follow up if it didn't get FDA approval… if it doesn't get approved, how much support are we really going to get?” (3F)

“So I don't know if Medtronic pays for it. I don't know if [the hospital] pays for it. I don't know if the government pays as part of the test. It's all very unclear to me to be honest with you...” (6F)

## Discussion

DBS researchers have long appreciated the significance of family members, yet attention has focused primarily on the material and psychosocial support that family members can provide [11, 128; 35]. While such support is indeed highly significant, our findings support and extend the view that family relationships influence (and are influenced by) DBS trials in ethically salient ways that go beyond these traditional forms of support.

One such ethically salient role that family members can play is the provision of what we call *epistemic support*. Recent qualitative studies of psychiatric DBS have shown that family members are uniquely positioned to provide clinically relevant insights about the effects of DBS, such as changes in psychiatric symptoms and side effects. Thomson et al. [27], for example, found that “spouses and close family members are likely to first notice any behavioral or psychiatric disturbances that can emerge after surgery and are key to treating them” (p. 245). Our study extends these recent findings about epistemic support: our interview data suggests that family members have the potential to not only provide clinically relevant insights that can help clinicians and patients learn about and understand DBS outcomes, but also that the *act of sharing* these insights with DBS recipients can actually influence the DBS outcomes themselves. This was the case when Participant 2 described his experience of learning about his family member’s insights as itself “healing.”

Though clinical researchers may already informally draw on family members in assessing outcomes and making clinical decisions, we argue that our findings justify formally expanding the traditional definitions of support to include epistemic support. Doing so would encourage the development of formal instruments for gathering and incorporating family member observations into clinical trials, thereby helping to meet the scientific goals of clinical trials and, in some cases, improve DBS outcomes. Such a move would also be in line with calls for increased emphasis on qualitative measures in regulatory standards governing DBS research (which currently, as Stevens and Gilbert [36] argue, largely prioritize quantitative measures), and also in line with “participants as partners” initiatives, which actively promote the inclusion of patients in health research beyond their traditional role as research participants [37]. Expanding traditional definitions of support would also have positive ethical implications. “Participants as partners” advocates argue that commitments to the values of integrity and respect for persons require that researchers recognize and value patient contributions to research [37]. A similar argument could be made with respect to recognizing family member contributions. Including epistemic support within definitions of support would facilitate the explicit recognition of the valuable epistemic work that family members perform and validate their contributions to the clinical trial team.

Although family involvement is widely understood to be significant in DBS trials, there are also concerns that family members can become *too* involved, exerting undue influence and threatening a patient’s autonomous decision-making [23]. The possibility for undue influence may be heightened in cases where individuals see DBS as their only remaining option for symptom relief and describe feelings of desperation and urgency, as some DBS recipients did in our study (see also Thomson et al. [28] and Klein et al. [22] for similar findings on patients' sense of desperation in decision-making about psychiatric DBS, yet see also Lawrence et al. [38] for evidence that patients with MDD are still able to participate in meaningful decision-making). While acknowledging the potential for undue influence, some bioethicists—especially those working from feminist, intercultural and non-Western traditions—have argued that these worries are frequently based on a misguided, Western-centric conception of autonomy as requiring independence from external influence [39–41]. In contrast, they argue that autonomy is best understood as a *relational* concept, and that freedom from external influence does not always equate to more autonomous decision-making [24, 42, 43].. Thus some “traditional efforts to ‘protect’ patients from their family or to override their decisions to involve their family” can in certain circumstances *reduce* rather than enhance patients’ autonomy [23, 132].

All participants in our study engaged in shared decision-making processes. Most participants discussed shared decision-making in positive terms, describing their family members as essential *partners* in the decision-making process, lending support to a relational model of autonomous decision-making. However, not all family member involvement led to more autonomous decision-making. One family member described what could be categorized as a case of undue influence: he explained that his perception of his wife’s severe symptoms led him to develop what he called “a nasty habit of translating for her” during informed consent discussions with the research team. Our results thus provide evidence for the view that what threatens autonomy is not only the *degree* of external influence, but also the *kind* [23]. In other words, it is not the case that greater family involvement in decision-making inevitably threatens autonomy (indeed, for most of our interviewees, greater family involvement was seen as supportive of autonomous decision-making).

Researchers have raised the concern that DBS may induce identity changes [20, 44–46]. Yet recent qualitative studies have found that risks of identity change were not a significant concern for individuals with psychiatric conditions considering DBS [47]. Further, others have pointed out that identity changes are not unique to DBS; identities are dynamic and shift in response to a variety of life events [48]. Nonetheless, researchers have also noted that adjustment to identity changes of any kind—even positive, “natural” changes—can be difficult [49] and often requires some identity “holding” by others [50, 51]. This study’s findings support the view that family members are vital in helping DBS recipients make sense of their new experiences and integrate them into their narratives of identity, and, further, that this is the case *even when* changes are anticipated and seen as positive.

Family relationships influence the process of DBS, and this process in turn influences relationships. When DBS successfully mitigated symptoms, this frequently improved relational dynamics by relieving family members’ responsibilities and allowing DBS recipients to take on new, meaningful relationship roles, such as becoming caregivers themselves, as was the case when Participant 1 became able to care for her aging partner. However, even when DBS was perceived to be successful, existing relationship tensions were sometimes replaced with new ones. The DBS itself was a conduit of some of these new tensions. DBS recipients were distressed when—after expressing negative emotions—their family members questioned whether their DBS was functioning. This experience highlighted a relational asymmetry introduced by DBS: as one DBS recipient described, when her husband becomes agitated, she cannot question the authenticity of *his* feelings by asking whether *his* DBS is on. These findings are in line with the results of a qualitative study in which one DBS recipient described how her father would suggest that clinicians should “turn her up” to make her feel better after a family fight, and another DBS recipient described it as “dehumanizing” to be asked if her device was on when she was acting differently than expected [22]. These results suggest that future study designs should consider not only the effects of DBS on recipients, but also the effects on family members and on the *relationships* themselves, and this is important even in cases where DBS outcomes are judged to be successful.

These findings raise a new question about experimental design: given the significance and complexity of family member roles in DBS trials, how can studies be designed to best support family members in taking on these roles? Many family members strongly value feeling included in DBS trials as part of a “team,” which suggests the need for including processes in study designs that acknowledge and facilitate robust, team-like involvement. To develop such processes, researchers could look to “participants as partners” initiatives, many of which discuss guidelines and best practices that could be extended to family members [37, 52, 53].

Bioethicists have called attention to ethical issues related to the post-trial period, such as determining the circumstances under which researchers have ethical obligations to provide continued access to care and maintenance of investigational brain implants or to cover the costs of explantation of the device [54–57]. Thus far, the focus has been primarily on the experiences and obligations of trial participants and clinical researchers, who have expressed significant concern and confusion about the post-trial period (see e.g. [58] and [55]. The findings from this study suggest that similar anxieties and confusions also weigh heavily on family members. Indeed, one family member (3F) believed that they were more concerned about financial obligations than the DBS recipient (3): “I always was a little hesitant… just wondering if we were going to be in this for a lot of money later, and it didn't affect our decision, but 3 was much less concerned”. These findings suggest that DBS research on post-trial obligations, including proposals for creating post-trial ethical standards and norms of practice (see e.g. [54]), should include family members alongside trial participants and clinical researchers as key stakeholders.

Finally, both DBS recipients and family members conceptualized DBS as one “tool” for treatment rather than a “cure.” Even participants who considered DBS to be highly successful understood symptom management to be an ongoing effort (see Versalovic et al. [59] for discussion of a similar theme with respect to the prospective use of DBS for substance use disorders). This is in line with the central findings of Thomson et al. [28], the only other study to date that we are aware of to include family members as interviewees: DBS recipients and family members in this study conceived of DBS as an ongoing “work in progress.” As such, the role that family members play in providing different forms of support, participating in decision-making, and adapting to effects such as identity change, will continue to be significant in the long-term regardless of DBS outcomes, providing additional justification for intentional and systematic integration of family member roles into study designs.

## Limitations

This study is retrospective: the interviews took place between 6 and 14 years after initial DBS implantation. As such, the results depend on recall, which “can be biased by current circumstances and interim events, leading to an inaccurate reflection of an individual’s actual experience” [60, 2218; 61]. On the other hand, as analysis revealed, some interviewees took extensive notes throughout the DBS process, thereby improving recall. Another limitation is that participants with more successful DBS outcomes may have been more able and inclined to participate in the study, resulting in possible underrepresentation of negative experiences within the data. In considering these limitations, it’s worth noting that this study—a qualitative study with a small number of participants—is not intended to be generalizable to all DBS recipients and family members across all contexts, even within psychiatric DBS. The aim of this study is to offer a nuanced, detail-oriented, textured analysis of particular experiences and perspectives.

## Conclusion

Family members are widely understood to provide essential support to individuals receiving medical care and participating in clinical trials [23, 62, 63]. This is very much the case for psychiatric DBS trials, where the participant population is categorized as “vulnerable” [64, 65]. Yet relatively little qualitative research exists on the roles that family members can and do play in these trials, on the kinds of support that they provide, or on the impacts of trial participation on family members and relationships. The findings from this study⁠—one of the first to include family members⁠—reveal how loved ones participate in psychiatric DBS trials in ways that extend beyond traditional conceptions of support provision. In turn, this study reveals the complex ways in which relationships can affect DBS experiences, and vice versa. Taken together, these findings suggest ways to improve study designs to better take into account relationships, and better support family members in taking on the complex, essential roles that they play in DBS trials for psychiatric conditions.

## Supplementary Information

Below is the link to the electronic supplementary material.

Supplementary file1 (DOC 68 kb)

## Data Availability

The participants of this study did not give written consent for their data to be shared publicly therefore supporting data is not publicly available.

## Footnotes

1 There are complex questions, which are beyond the scope of this paper, regarding how to define and measure success in psychiatric DBS trials [see 21]. We asked recipients if they viewed DBS as a success and responses were judged qualitatively based on a scale of ‘Highly successful’ to ‘Unsuccessful.’ See Table 1.
