# Supplementary material for: The Role of Family Members in Psychiatric Deep Brain Stimulation Trials: More Than Psychosocial Support
Source: Neuroethics. 2023 May 26;16(2):14. doi: 10.1007/s12152-023-09520-7 (PMC10212803; doi:10.1007/s12152-023-09520-7)
Supplement: Supplementary file 1 — Supplementary file1 (DOC 68 kb) [file 12152_2023_9520_MOESM1_ESM.doc]

# Supplementary Information: Interview Guide for DBS Recipients

**I. Introduction and Warm-Up**

*Instructions for Interviewer: Begin by introducing yourself and reminding the interviewee of the consent process and that they are free to end the interview at any time and/or to skip any questions they’d prefer not to answer.*

- You have been invited to give your perspective on using DBS for psychiatric illness, particularly as it pertains to your relationships with caregivers and loved ones. If it’s alright with you, I’m going to ask you a number of questions to get a better sense of your experiences and thoughts.
- Tell me a bit about yourself, and your life with mental illness prior to the DBS.
  - What were the challenges?
  - What other treatments had you undergone prior to DBS?
- How long have you had your device? Does it allow for any kind of control by you? If so, what kind of control?

**II. Awareness/Understanding**

# How did you first hear of DBS as a treatment option?

- Were any aspects of DBS confusing to you or hard to understand?

**III. Informed Consent and Decision-Making**

# How did you decide to go forward with DBS treatment?

# What affect (if any) did family members[[1]](#footnote-2) have on your decision-making process regarding DBS?

# To whom (if anyone) did you consult/ask for advice in deciding to use DBS? What did they say? In what ways was it helpful or unhelpful?

- Was there anything you feel that you did not understand at the time, or wish had been included, in the consent process?

# IV. Attitudes Towards DBS

# What did you think when the possibility of DBS was initially raised?

- What did your family member think when the possibility of DBS was initially raised?

**V. Expectations**

# What did you expect would happen as a result of DBS?

# *Prompt*: Best case? Worst case?

# What happened that you did or didn’t expect?

# What surprised you? What was unusual?

# (*Alt:* What experiences stand out to you following the start of DBS treatment?)

- What has been the hardest thing about DBS? Did you expect this to be hard?
- What has been the best/easiest thing about DBS? Was this surprising?
- What do you think your family members were expecting regarding DBS?

**VI. Relationship Effects, Identity and Agency**

- What role (if any) have your family members played in your life since you received the DBS?
- In what ways (if any) do you feel different since you received the DBS?
  - Has your sense of self changed at all after getting the DBS? If so, how?
  - *Alternative:*  Have you noticed any change in yourself from before the surgery? How would you describe that change? (Was it positive/negative overall? Intended or not? Etc.)
- Have others commented on any changes in you since you received the DBS? If so, what have they noticed?
  - *Prompt:* Any changes in your personality or way of looking at things?
  - *Follow up:* Do you agree with what they say about how you’ve changed? If so, why? If not, why not?

# Do you feel that your relationships changed or stayed the same since you received the DBS? How so?

# What did you hope would happen to the relationship between you and your family members since you received the DBS?

- Do you feel that the DBS has changed your sense of agency or the ability to do what you want to do? If so, how?
- Do you think that you or your family member is better able to tell if the DBS is working well? Can you explain?
  - *Follow up:* If you can adjust the settings on your DBS (and if you can’t, imagine if you could), who do you think is in the best position to know when to make changes?
- To what extent are you (or would you be) comfortable with your family members giving input on how your DBS stimulation levels might need to be changed?
  - *Follow up:* If your family member has given such input in the past, how have you responded?

# Has getting a DBS changed the way you interact with your health care provider(s)?

- - *Prompt:* Affect on trust? Reliance? Dependence?
- Do you think your family member’s role in this treatment is different from the role they played in other treatments? If yes, how so?

# *Alternative phrasing:* Has the fact that you received a DBS given them a better or more immediate way to influence your treatment?

# *Prompt:* Has getting a DBS changed the way your family member interacts with your health care provider(s)?

# In prior work, some DBS users have mentioned that they can feel like their family members seem to think they can be controlled by the device, or that they sometimes question whether their thoughts and feelings are “real”. Have you had that experience or experienced anything like that?

# 1. After getting the device, have you had a time when you wondered whether something you were feeling or thinking was artificial or not really yours in some way?

# 2. After getting the device, do you feel like you remain in control of your thinking or feeling, or do you ever feel less fully in control? If so, can you explain how, or when that happens?

# 3. Would you say that the device has helped you to be “true to yourself”? Or has it made being yourself difficult in any way? (For either, can you explain what you mean, or how you have felt?)

# *Alternative:* How do you talk about the implant? how does it come up? do people in your family ever attribute actions to the implant or lack of stimulation?

#

# VII. Concluding Questions

# Has DBS been beneficial? What role do you think your family members played in this outcome?

- - What were some of the most helpful/beneficial ways in which family members affected your experience as a DBS user? Why?
  - What were some of the most unhelpful ways in which family members affected your experience as a DBS user? Why?

# Knowing what you know now about DBS, what (if anything) would you change regarding your relationships with family members (e.g. would you ask them to be more or less involved? To come to more appointments or fewer?, etc)?

- Looking back, do you think that getting a DBS was the right decision? Why or why not?

# Supplementary Information: Interview Guide for Family Members

**I. Introduction and Warm-Up**

*Begin by introducing yourself and reminding the interviewee of the consent process and that they are free to end the interview at any time and/or skip any questions they’d prefer not to answer.*

- You have been invited to give your perspective on caring for, or living with, a person who has received DBS for depression or OCD. If it’s alright with you, I’m going to ask you a number of questions to get a better sense of your experience and your perspectives on DBS.
- First, if you’re comfortable talking about it, I’d like to hear a little about your experience with your family member[[2]](#footnote-3) who has been living with mental illness prior to DBS.
- What other treatments has your loved one tried and what was your involvement in those treatments?
- How long ago did your family member get a DBS? Does it allow for any at-home control or adjustments of settings? If so, what kind of control?

**II. Awareness/Understanding**

# Tell me about how you first heard about DBS as a treatment option for your family member.

- Were any aspects of DBS confusing to you or hard to understand?

**III. Informed Consent and Decision-Making**

# What (if anything) was your involvement in the patient’s decision to use DBS?

# Did you participate in this patient’s informed consent process? If so, how?

# What (if anything) do you feel that you did not understand at the time, or wish had been included, in the consent process?

# IV. Attitudes Towards DBS

# What did you think when DBS was first proposed as a treatment for your family member?

# What did your family member think?

**V. Expectations**

# What did you expect would happen as a result of DBS?

# *Prompt*: Best case? Worst case

# What happened that you did or didn’t expect?

# What surprised you? What was unusual?

# (*Alt:* What experiences stand out to you following DBS surgery?)

- What was the hardest thing about DBS treatment? Did you expect this to be hard?
- What has been the best/easiest thing about DBS treatment? Was this surprising?
- What do you think your family member was expecting regarding DBS?

**VI. Relationship Effects, Identity and Agency**

- How have you participated in the patient’s life following DBS surgery?
- Do you think that your loved one has changed at all since the surgery? If so, how?

# W ould you say that your loved one’s sense of self changed at all after getting the DBS? If so, how?

# *Prompt:* Have you noticed any changes in their personality or way of looking at things?

# *Alternative:*  "Have you noticed any change in your love one from before the surgery? How would you describe that change? (Was it positive/negative overall? Intended or not? Etc.)

# Has your loved one mentioned to you any changes that they notice about themselves? If so, what have they mentioned?

# Do you agree with what they say about how they’ve changed? If so, why? If not, why not?

# Do you feel that your relationship changed or stayed the same following DBS surgery? How so?

# What (if anything) did you or your family member hope would happen to the relationship between the two of you?

# Do you feel that the DBS has changed your loved one’s sense of agency or the ability to do what they want to do? If so, how?

# Do you think that either you, or your loved one, is better able to tell if the DBS is working well? Can you explain?

# If your loved has a DBS with self-adjustable settings (or, if not, imagine that they do), who do you think is in the best position to know when to make changes?

# To what extent are you/would you be comfortable with giving input to your family member on how their DBS stimulation levels might need to be changed?

# If you have given such input in the past, how has your loved one responded?

# Do you think your role in your loved one’s DBS treatment is different from the role you played in other treatments? If yes, how so?

# *Altnerative phrasing:* Has the fact that your family member uses DBS given you a better or more immediate way to influence their treatment?

# *Prompt:* Has getting a DBS changed the way you interact with you family member’s health care provider(s)?

# *Possible follow-up:* In prior work, some DBS users have mentioned that they can feel like their family members seem to think they can be controlled by the device, or that they sometimes question whether their thoughts and feelings are “real.” What do you think about that? Does it make sense to you?

**VII. Concluding Questions**

# If your loved one benefitted from DBS, what role do youthink you played in this?

# Looking back, do you think DBS was the right decision for your family member? Why or why not?

# What (if anything) would you do differently if you were starting the DBS process again, but knowing what you now know about it?

1. NOTE: the term ‘family member’ here is used to represent a person with whom the DBS user has an important relationship. The relevant term or terms (e.g. spouse, child, friend, loved one, caregiver) will be chosen based on the specific details of each interview. [↑](#footnote-ref-2)
2. NOTE: the term ‘family member’ here is used to represent the person who uses DBS. The relevant term or terms (e.g. spouse, child, friend, loved one, caregiver) will be chosen based on the specific details of each interview. [↑](#footnote-ref-3)
